# Women's academic leadership and the governance of digital public health systems: an opinion

**DOI:** 10.3389/fpubh.2026.1792961

**Published:** 2026-04-10

**Authors:** Muthu Kumari, Bini Marin, R. Sujithra, B. Sreya

**Affiliations:** Joy University, Kanyakumari, India

**Keywords:** AI ethics, digital public health governance, gender equity, higher education, public health informatics, women's academic leadership

## 1 Introduction

Digital public health is increasingly recognized as a core pillar of population health systems, encompassing digital surveillance, artificial intelligence (AI), data governance, and technology-enabled decision-making for disease prevention and health promotion (1). Academic institutions play a central role by producing evidence, training the public health workforce, and advising governments and international agencies. Leadership within academia significantly influences how digital public health systems are conceptualized, governed, and implemented. Women make up a substantial proportion of the global public health and health sciences workforce, yet they remain underrepresented in senior academic and institutional leadership positions (2, 3). Recent global analyses indicate that women represent nearly 70% of the global health workforce but occupy only about 25%−30% of senior leadership positions in health-related academic and research institutions (4). More recent analyses further show that women account for less than 30% of full professors globally and fewer than 20% of university vice-chancellors or presidents, indicating persistent vertical segregation in academic leadership (5). Similar patterns have been observed across biomedical and public health disciplines where women's representation declines substantially at higher academic ranks such as dean, director, and department chair positions (6). This pattern is often explained through the “glass ceiling” and “leaky pipeline” frameworks, which describe structural and cumulative barriers that limit women's progression into senior leadership despite comparable qualifications (7). This imbalance is not only a gender equity issue but also affects institutional capacity, ethical governance, and responsiveness to population health needs. Leadership decisions shape research priorities, resource allocation, and engagement with public policy, all of which are critical for developing effective digital public health systems (8). From a health systems governance perspective, leadership diversity is increasingly recognized as a structural determinant of institutional innovation, ethical decision-making, and responsiveness to population health needs. This opinion article proposes that women's academic leadership is an important factor shaping digital public health capacity. Drawing on evidence from public health, digital health equity, and higher education research, it argues that inclusive leadership strengthens governance, workforce development, and ethical oversight. This argument is further supported by intersectionality theory, which emphasizes how overlapping social identities including gender shape access to power and decision-making within institutions. The discussion is globally relevant and particularly salient for low- and middle- income countries, where leadership capacity strongly influences public health preparedness and resilience. Emerging research on global health governance further indicates that leadership diversity contributes to improved institutional decision-making, ethical oversight, and innovation in health systems (9). In the context of digital public health, inclusive leadership has been shown to influence how AI-enabled systems are developed in ways that promote equity, transparency, and public trust.

## 2 Gender inequities in academic leadership: implications for public health systems

Gender disparities in academic career progression are well documented worldwide. Despite comparable qualifications and research productivity, women remain underrepresented in senior academic and decision-making roles (10). Large-scale studies of biomedical and public health institutions demonstrate that women hold fewer than one-third of senior leadership roles globally despite substantial representation among early-career researchers. Structural barriers including implicit bias, unequal access to professional networks, and disproportionate teaching and service responsibilities continue to influence leadership trajectories in academia (11). Leadership selection processes often rely on informal networks and linear career expectations, disadvantaging non-traditional trajectories and reinforcing cumulative disadvantage over time. Women faculty members are also more likely to assume teaching, mentoring, and service responsibilities that are essential to institutional functioning but undervalued in promotion and leadership criteria (12). In public health education and research, these patterns have practical consequences. Academic leaders influence curriculum design, prioritization of digital competencies, and engagement with national and international public health systems. Underrepresentation of women in these roles may limit the diversity of perspectives shaping digital public health agendas and governance structures (13). From a public health systems perspective, leadership inequities can affect population health outcomes. Decisions regarding AI-enabled surveillance tools, digital data governance, and responses to health misinformation are influenced by institutional leadership cultures. A lack of diversity in leadership risks narrowing technological priorities and insufficiently addressing equity, trust, and social determinants of health, which are foundational principles of public health. Empirical evidence from global health leadership studies indicates that gender-diverse leadership teams are associated with improved organizational performance, stronger community engagement, and more inclusive policy design, particularly in health systems (14). Recent digital health governance research further suggests that leadership diversity is associated with improved institutional responsiveness to equity concerns, community engagement, and ethical oversight in technology-enabled public health systems (15).

## 3 Digital public health governance and academic leadership

Digital public health extends beyond technology to include governance, ethics, and accountability. Ethical challenges associated with AI-enabled public health systems, such as algorithmic bias, transparency, and data protection, require inclusive and interdisciplinary oversight (16). Academic leaders play a key role in shaping institutional responses to these challenges through research leadership, policy engagement, and workforce training. Evidence from digital health equity research suggests that leadership diversity contributes to anticipatory governance and greater attention to equity risks in system design (15). Evidence suggests that women leaders are more likely to emphasize participatory research, ethics, and community engagement, essential for responsible digital public health implementation. Their underrepresentation may constrain the ethical and social responsiveness of digital public health systems. Academic institutions also act as intermediaries between technological innovation and public health practice. Leadership decisions influence partnerships with surveillance agencies, investment in public health informatics infrastructure, and the integration of digital competencies into curricula. Inclusive leadership is therefore central to building sustainable digital public health capacity. This issue is particularly relevant in low- and middle-income countries (LMICs), where academic institutions often play a central role in workforce training, digital health research, and policy advisory functions. LMICs continue to experience a disproportionate burden of infectious diseases, maternal mortality, and emerging health threats while simultaneously undergoing rapid digital health expansion (17). For instance, LMICs account for approximately 94% of global maternal deaths and face persistent gender disparities in access to healthcare, digital technologies, and health information systems (18). In addition, the digital gender gap remains significant, with women in LMICs being 19% less likely than men to use mobile internet, limiting their participation in digital health ecosystems (19). Gender disparities in access to healthcare, maternal health outcomes, and representation within the health workforce further highlight the importance of inclusive leadership in shaping equitable digital public health systems (20). These challenges underscore the need for women's leadership in academic public health institutions, as gender-responsive leadership is more likely to prioritize maternal health, community-based surveillance, and inclusive digital health interventions tailored to vulnerable populations. Recent evidence indicates that women remain underrepresented in academic leadership across LMICs. Studies from South Asia and Sub-Saharan Africa show that women occupy less than 25% of senior academic leadership positions in health and public health institutions, despite constituting a substantial proportion of the workforce (21). In India, for example, women represent approximately 43% of faculty in higher education but remain significantly underrepresented in senior administrative and decision-making roles (22). These disparities highlight structural constraints in LMIC academic systems that directly influence leadership pipelines and decision-making in digital public health governance. Inclusive academic leadership can influence how digital surveillance systems, health data governance frameworks, and public health informatics training programs address gender-sensitive health priorities and promote equitable access to digital health innovations (29).

## 4 Digitally enabled leadership development as capacity building

Digital platforms have transformed professional collaboration, mentoring, and leadership development in academia. Digitally mediated mentoring and leadership programs offer scalable strategies for strengthening institutional capacity. Virtual mentoring initiatives can reduce geographic and institutional barriers, enabling women academics to access senior leadership guidance and global professional networks (23). Structured leadership development programs delivered online or in blended formats are associated with improved leadership readiness, strategic capacity, and professional confidence (24). Recent evidence from global mentorship programs in public health further demonstrates that structured digital mentoring significantly improves leadership advancement and research productivity among women academics in LMIC settings (25). When combined with institutional recognition and accountability mechanisms, these initiatives support transparent and inclusive leadership pathways. However, digital approaches are not inherently equitable. Without explicit governance and monitoring, digital systems may reproduce existing power imbalances or exacerbate disparities (26). Gender- disaggregated institutional data systems, long used in public health monitoring, can track recruitment, promotion, workload distribution, and leadership participation, reinforcing evidence- informed governance. Recent research also highlights that digitally enabled professional networks and mentoring initiatives can strengthen leadership pipelines for women academics by facilitating access to international collaborations and leadership training opportunities (31).

## 5 Discussion

This Opinion highlights women's academic leadership as an under-recognized factor shaping digital public health capacity. While digital technologies offer powerful tools for disease surveillance, emergency response, and health promotion, their effectiveness depends on governance structures and leadership decisions. However, the relationship between leadership diversity and digital public health outcomes requires stronger conceptual grounding. Leadership in academic institutions influences which health priorities are emphasized, how digital tools are designed, and how ethical safeguards are implemented. Studies in global health governance indicate that diverse leadership teams are more likely to incorporate equity-focused approaches, participatory decision-making, and community engagement into health systems (29). For example, emerging evidence from global health programs suggests that gender-diverse leadership teams contribute to more inclusive digital health interventions and improved community engagement outcomes. Persistent gender inequities in academic leadership may limit the ethical, equitable, and socially responsive development of digital public health systems. Digital innovation alone cannot dismantle entrenched institutional norms (30). Sustainable progress requires alignment between digital strategies and structural reforms addressing promotion criteria, workload distribution, and leadership recognition. This issue is particularly critical in low- and middle-income countries (LMICs), where public health systems face intersecting challenges such as high maternal mortality, infectious disease burden, and limited digital infrastructure. Evidence shows that LMICs continue to bear a disproportionate burden of maternal mortality and health system challenges (27). In such contexts, inclusive academic leadership can influence the prioritization of gender-responsive digital health interventions, including maternal health surveillance systems, community-based data collection, and equitable access to digital health services. Similar to population health monitoring systems, academic institutions can apply gender- disaggregated monitoring frameworks to track leadership participation, promotion patterns, and institutional equity outcomes. Such monitoring enables evidence-informed governance, improves accountability, and helps institutions identify structural barriers affecting women's progression into leadership roles. Global policy analyses emphasize that digital transformation must be accompanied by robust governance and accountability mechanisms to achieve equity and long-term impact (28). Applying public health governance principles such as surveillance, monitoring, and evaluation to academic leadership structures provides a logical and evidence-based approach to strengthening institutional accountability and ensuring that leadership diversity translates into improved digital public health outcomes. Higher education institutions are encouraged to integrate gender equity objectives into digital public health training, AI governance initiatives, and institutional data systems. Recognizing diverse career trajectories and valuing contributions to mentoring, service, and public engagement are essential for inclusive leadership development. Advancing women's academic leadership is therefore both a matter of fairness and a strategic investment in digital public health resilience and population health outcomes. Strengthening women's leadership representation in academia may therefore enhance institutional capacity to address emerging digital public health challenges including ethical AI governance, digital misinformation, and equitable access to health technologies.

## 6 Conclusions and recommendations

Women's academic leadership should be viewed as a strategic component of digital public health systems rather than a peripheral equity concern. Academic leaders shape research agendas, governance frameworks, and workforce development pathways that determine how digital public health tools are designed, implemented, and evaluated. Persistent gender imbalances in leadership have implications for ethical governance, inclusiveness, and effectiveness. Strengthening women's leadership contributes to more resilient and socially responsive digital public health ecosystems. Inclusive leadership enhances attention to equity, transparency, and community engagement, principles central to public health practice and increasingly important in AI-enabled, data-intensive environments. Aligning leadership development with digital public health principles offers a practical pathway toward more equitable, accountable, and effective population health systems. Addressing gender disparities in academic leadership is integral to advancing ethical innovation, workforce sustainability, and improved population health outcomes. Recognizing women's academic leadership as a strategic component of digital public health governance can therefore contribute to more inclusive, resilient, and ethically grounded health systems in an increasingly data-driven global health landscape.

### 6.1 Recommendations

a) Higher education institutions should integrate gender equity objectives into digital public health strategies, including AI governance frameworks, surveillance partnerships, and informatics programs. This can be operationalized by conducting institutional gender audits, embedding gender-responsive AI ethics modules within public health curricula, and establishing measurable indicators (e.g., proportion of women in digital health leadership roles) that are monitored annually.
b) Digitally enabled mentoring and leadership development initiatives should be institutionalized and recognized within promotion and leadership criteria, through structured digital mentoring networks, virtual leadership training programs, and international academic collaboration platforms. This may include creating formal e-mentorship platforms, pairing early-career women academics with senior leaders, integrating mentorship outcomes into promotion criteria, and supporting cross-institutional and international leadership fellowships using digital platforms.
c) Universities should adopt gender-disaggregated data systems to monitor recruitment, promotion, workload allocation, and leadership participation, applying public health monitoring principles to academic governance in order to strengthen evidence-based institutional decision-making and accountability. This can be implemented by developing institutional dashboards that track gender-based metrics (recruitment, promotion rates, pay equity, leadership roles), conducting periodic audits, and publicly reporting findings to enhance transparency and accountability.
d) Policymakers and funding agencies should recognize leadership diversity as a key component of digital public health capacity building by encouraging gender- balanced leadership teams in digital health research initiatives. This can be achieved by incorporating gender equity criteria into grant evaluation frameworks, prioritizing funding for projects with diverse leadership teams, and supporting targeted leadership development programs for women in digital public health.

Furthermore, integrating public health monitoring principles into academic governance is essential because it enables continuous evaluation, data-driven decision-making, and accountability, which are core components of effective public health systems that can be directly applied to leadership governance in higher education.
